# Maintenance therapy of low-dose nivolumab, S-1, and leucovorin in metastatic pancreatic adenocarcinoma with a germline mutation of *MSH6*: A case report

**DOI:** 10.3389/fimmu.2022.1077840

**Published:** 2022-12-13

**Authors:** Shang-Hsuan Peng, Bang-Bin Chen, Ting-Chun Kuo, Jen-Chieh Lee, Shih-Hung Yang

**Affiliations:** ^1^ Department of Oncology, National Taiwan University Hospital, Taipei, Taiwan; ^2^ Department of Oncology, National Taiwan University Hospital Yunlin Branch, Yunlin, Taiwan; ^3^ Department of Medical Imaging and Radiology, National Taiwan University Hospital, Taipei, Taiwan; ^4^ Department of Traumatology, National Taiwan University Hospital, Taipei, Taiwan; ^5^ Department of Pathology, National Taiwan University Hospital, Taipei, Taiwan; ^6^ Graduate Institute of Oncology, National Taiwan University College of Medicine, Taipei, Taiwan

**Keywords:** pancreatic ductal adenocarcinoma, nivolumab, maintenance therapy, mismatch repair, case report

## Abstract

Immune checkpoint inhibitors (ICIs) provide substantial benefits to a small subset of patients with advanced cancer with mismatch repair deficiency (MMRD) or microsatellite instability (MSI), including patients with pancreatic ductal adenocarcinoma (PDAC). However, the long duration of ICI treatment presents a considerable financial burden. We present the case of a 63-year-old woman with metastatic PDAC refractory to conventional chemotherapy. Genetic analyses identified an *MSH6* germline mutation and a high tumor mutation burden (TMB). Complete response (CR) was achieved after a short course of low-dose nivolumab (20 mg once every 2 weeks) with chemotherapy. CR was maintained for over 1 year with low-dose nivolumab and de-escalated chemotherapy without any immune-related adverse events. This case supports the further exploration of low-dose, affordable ICI-containing regimens in patients with advanced MSI-high/TMB-high cancer.

## Introduction

Pancreatic ductal adenocarcinoma (PDAC) is a malignancy with poor prognosis, and there has been little progress in the development of novel therapeutics for its treatment. Standard systemic therapy, comprising gemcitabine-based or 5-fluorouracil, leucovorin, irinotecan, and oxaliplatin (FOLFIRINOX)-like regimens, is the recommended first-line chemotherapy for metastatic PDAC with good performance status (PS). These regimens have been the standard for more than 10 years (1). Beyond progression under frontline gemcitabine-based therapy, a combination of nanoliposomal irinotecan, fluorouracil, and leucovorin (NaFL), which was tested in the NAPOLI-1 trial, has demonstrated marginal efficacy in terms of response rate (RR) and survival (1). According to data from randomized trials and real-world data, the clinical benefits decrease with successive lines of chemotherapy. Moreover, given its considerable toxicity, it may not be justified to administer further multiagent chemotherapy to patients with deteriorating PS beyond first-line chemotherapy.

In the past 10 years, immunotherapies based on immune checkpoint blockade with manageable toxicities have revolutionized the landscape of anticancer treatment, particularly for refractory solid tumors other than PDAC. Single-agent or combination immune checkpoint inhibitors (ICIs) have exhibited poor RR and survival in advanced PDAC, even when used in conjunction with chemotherapy (2). Nevertheless, tumors associated with DNA mismatch repair deficiency (MMRD) and characterized by microsatellite instability (MSI) and high tumor mutation burden (TMB) represent a small subset (~1%) of PDAC with high RR and long duration of response (DOR) to ICIs (3, 4). Although they provide considerable benefits for a subgroup of patients, the optimal dosing, timing, and combination of ICIs are unknown, and their financial burden is high.

Herein, we report a case of metastatic *MSH6*-mutated PDAC refractory to standard frontline palliative chemotherapy regimens indicating a complete and durable response achieved by low-dose nivolumab plus chemotherapy.

## Case presentation

A 63-year-old woman initially presented with intermittent periumbilical pain for half a year. She had undergone resection and adjuvant chemotherapy for stage I ovarian micropapillary serous carcinoma at the age of 52. In addition, she had a thoracic spinal epidural schwannoma that had been resected at the age of 61. Her sister had metachronous endometrial cancer and breast cancer in her 60s, and her father had lung cancer. Abdominal magnetic resonance imaging revealed an infiltrative hypoenhancing tumor measuring 2.2 cm in diameter at the pancreatic head. She underwent the Whipple procedure in September 2019, and the pathology report indicated pT2N0 stage IB poorly differentiated PDAC. Because of the cancer history of the patient and her family, genetic tests were recommended. Germline testing of a blood sample revealed a heterozygous mutation of the *MSH6* gene [c.3018C>G (p.Tyr1006Ter)]. The tumor tissue panel revealed the same *MSH6* mutation, heterozygous deletion of the *MLH1* gene, and additional genetic alterations (**Supplementary Table 1**). The tumor was MSI-high with a TMB of 52.8 mutations per megabase. Immunohistochemistry (IHC) revealed complete loss of MSH6 expression but a weak and heterogeneous expression of MLH1 in the neoplastic ducts. The expression of MSH2 and PMS2 was preserved (**Supplementary Figure 1**).

Subsequently, six monthly cycles of adjuvant gemcitabine and tegafur/gimeracil/oteracil (S-1) were administered. Recurrence with peritoneal metastases was noted soon after completion of adjuvant chemotherapy with doubling of the cancer antigen 19-9 (CA 19-9) level. One cycle of gemcitabine with nab-paclitaxel was administered. However, chemotherapy was temporarily withheld for the treatment of cryptococcal pneumonia. The level of CA 19-9 rapidly increased during the 4-month chemotherapy-free period. After the successful treatment of the infection, palliative chemotherapy was changed to NaFL.

**Figure 1:**
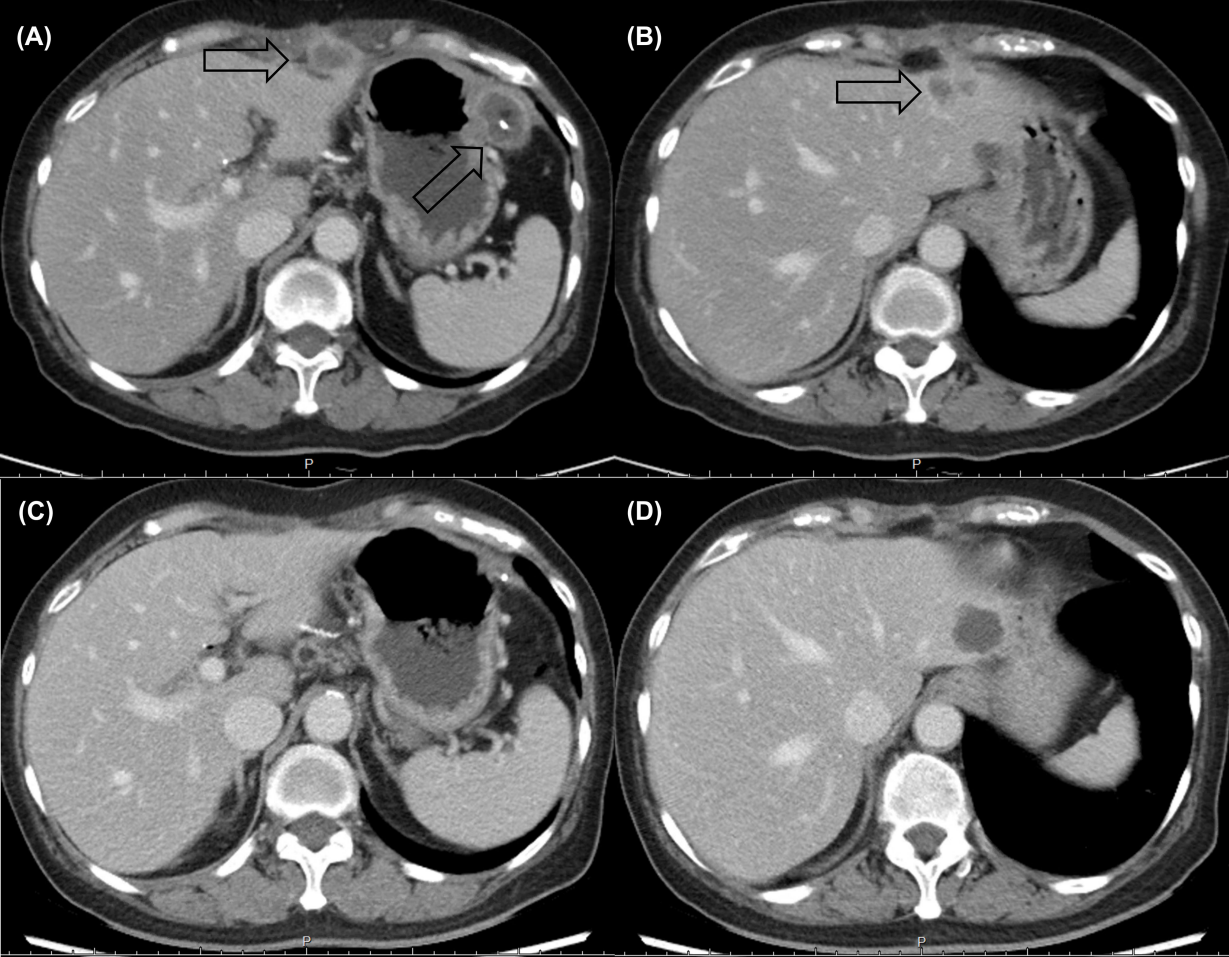
Computed tomography revealed multiple rim-enhancing **(A)** peritoneal metastases and **(B)** liver metastases before low-dose nivolumab. A durable complete response (**C**, **D**) was maintained after more than 1 year of nivolumab-based treatment.

**Figure 2:**
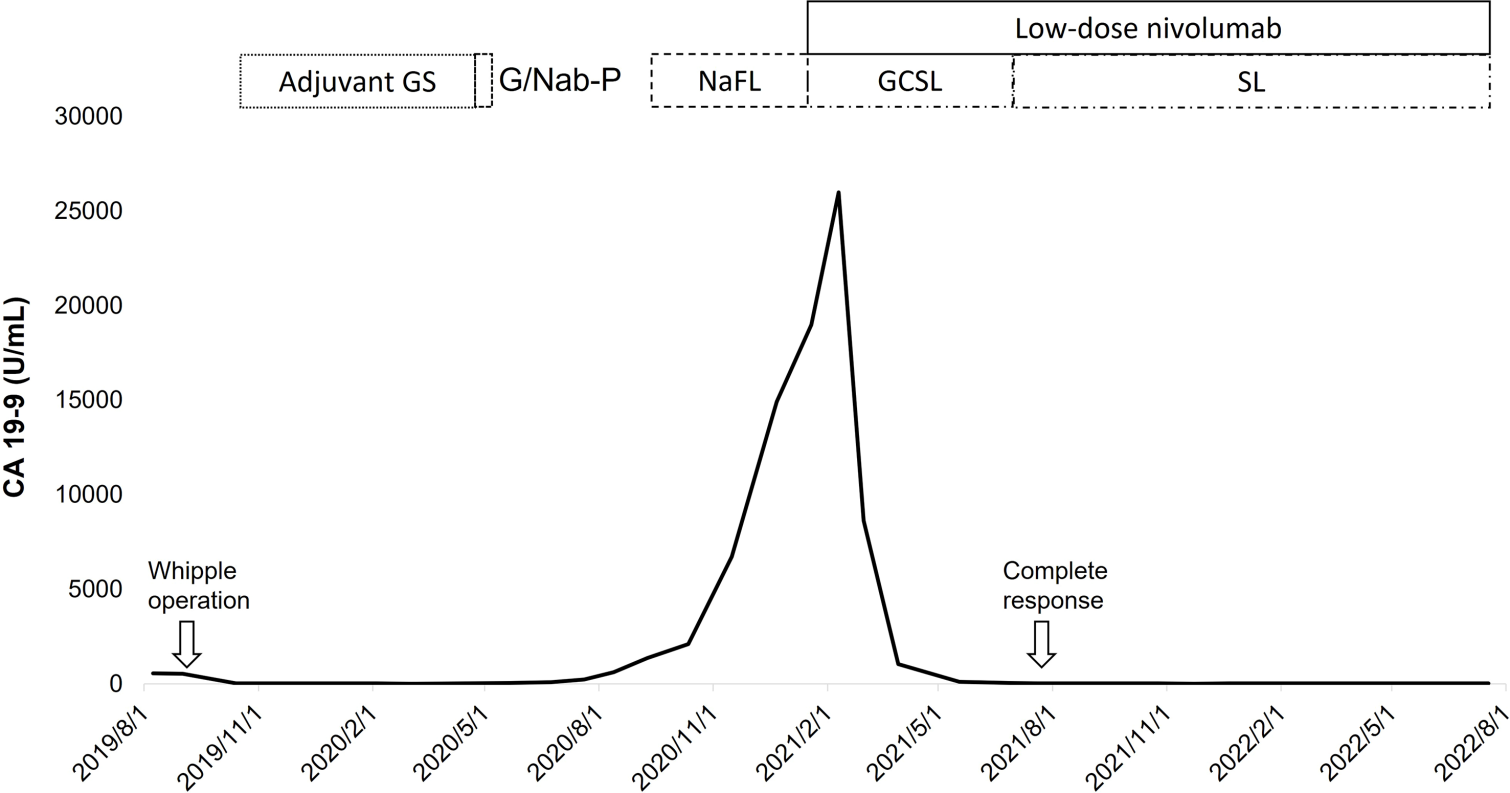
With a short duration of low-dose nivolumab plus gemcitabine (G), cisplatin (C), S-1 (S), and leucovorin (L), the level of CA 19-9 decreased rapidly with a complete response of tumors, which was durable under the maintenance therapy with low-dose nivolumab plus de-escalated chemotherapy (S-1 and leucovorin). (F, fluorouracil; Na, nanoliposomal irinotecan; Nab-P, nab-paclitaxel).

Following eight cycles of NaFL, peritoneal metastases progressed with new liver metastases (**Figures 1A, B**). Based on the results of genetic tests, standard-dose anti-programmed cell death 1 (anti-PD-1) therapy was recommended, but this treatment was not affordable for the patient. With the approval of the patient, a biweekly low dose of nivolumab (0.3 mg/kg, 20 mg) combined with cisplatin (40 mg/m^2^), gemcitabine (500 mg/m^2^), S-1 (20 mg bid), and leucovorin (15 mg bid) was started in February 2021. After six cycles of nivolumab with chemotherapy, in April 2021, computed tomography revealed marked tumor reduction. Because of cisplatin-associated renal dysfunction and extreme tumor reduction, nivolumab, S-1, and leucovorin have been administered without gemcitabine and cisplatin since July 2021. With ongoing low-dose nivolumab plus S-1 and leucovorin for more than 1 year, CR has been maintained (**Figures 1C, D**), and she has remained asymptomatic with gradually recovered renal function. The treatment course is summarized in **Figure 2**.

## Discussion

Maintenance therapy in advanced malignancies is understudied in cancer types with poor RR and short progression-free survival (PFS) with chemotherapy, such as PDAC. In the largest prospective study on PDAC, the PANOPTIMOX-PRODIGE 35 trial, the comparable median PFS and overall survival (OS) between a maintenance LV5FU2 regimen following disease control with eight cycles of FOLFIRINOX and 12 cycles of FOLFIRINOX in the first-line setting were demonstrated (5). However, patients may not recover from the toxicity of chemotherapy, such as the neurotoxicity of oxaliplatin; toxicity may even progress further as a result of restarting the same regimen (5). Sunitinib, a multitargeted receptor tyrosine kinase inhibitor, may be an acceptable alternative, although inadequate because of the limited benefit to PFS reported in the PACT-12 trial (6).

By contrast, meaningfully prolonged PFS has been achieved with maintenance olaparib, a poly(ADP–ribose) polymerase inhibitor, in metastatic PDAC with germline *BRCA1* or *BRCA2* mutations (7). However, the financial burden of olaparib may preclude the recommendation of olaparib in daily practice among the small group of patients because no OS benefit was reported in the POLO trial (7).

The safety and efficacy of pembrolizumab, an anti-PD-1 antibody, has been well documented in patients with MMRD (4). In the pancreatic cancer subgroup of the KEYNOTE-158 study utilizing pembrolizumab at 200 mg once every 3 weeks, the median DOR was 13.4 (8.1 to 16.0+) months (4). However, the financial burden of prolonged use is even greater than that of olaparib, and this may limit access to full-dose anti-PD-1 therapy in low- and middle-income populations. However, in the phase I trial of nivolumab monotherapy, the plateau and dynamic levels of PD-1 occupancy on the circulating CD3^+^ lymphocytes were similar among dose levels ranging from 0.3 to 10 mg/kg (8). The *in vitro* nivolumab concentration of 0.04 µg/mL could occupy >70% PD-1 on T cells, and pharmacodynamic tests indicated sufficient and durable PD-1 blockade at a low serum level (8, 9). Furthermore, the RR was not correlated with the dose of nivolumab; this provides ethical and scientific support for the application of low-dose nivolumab. For tumor-infiltrating lymphocytes (TILs), considerably more CD8+ TILs were observed in patients with MMRD PDAC (all with *MSH6* loss) compared to those without (median, 626 *vs.* 124 cells/mm^2^) (10). Therefore, a high number of neoantigen-specific TILs in patients with MMRD PDAC is expected and may largely compensate for the potentially inferior efficacy of nivolumab at even low doses.

Regarding our case, the history of multiple malignancies in her family and the genetic analyses of blood and tumor tissue were consistent with the presence of MMRD. IHC confirmed the loss of MSH6 expression. The heterogeneous and weak expression of MLH1 may probably reflect the heterogeneity of the promoter hypermethylation and also partially contribute to the high TMB in tumor cells. Short disease-free survival after adjuvant chemotherapy and rapid progression under third-line NaFL indicated limited treatment options and poor survival from further palliative chemotherapy if she had PDAC that was MMR-proficient or without actionable genetic alterations, such as *KRAS* G12C or *BRCA* mutations. Although the 2.1-month median time (range: 1.3–10.6) to response reported in the KEYNOTE-158 study was similar to that of fourth-line gemcitabine, cisplatin, S-1, and leucovorin in our case (4), the possibility of cytoreduction or enhancement of the antitumor immune responses from chemotherapy cannot be excluded. Because of the heavily pretreated status, considerable tumor burden, and uncertain efficacy of the low-dose nivolumab in our case, the administration of chemoimmunotherapy was reasonable to maximize the chance of disease control.

A previous pilot study, exploring cisplatin plus S-1 in pancreatic cancer patients who had failed postoperative gemcitabine, demonstrated RR of 29.4%, stable disease of 11.8%, and median OS of 10 months (11). Regarding the disease-free survival of more than 6 months with adjuvant gemcitabine plus S-1 in this patient and the activity of cisplatin plus S-1, the application of gemcitabine, cisplatin, low-dose S-1, and leucovorin was a reasonable and feasible chemotherapy backbone for the heavily pretreated patient. However, the timing of anti-PD-1 therapy in patients with MMRD PDAC remains undetermined. In the KEYNOTE-177 study on metastatic colorectal cancer with MMRD, the non-significant difference reported in median OS between the first-line pembrolizumab and chemotherapy was probably due to the crossover to PD-1 pathway blockade in 60% of the chemotherapy arm; this also reflects the uncertainty regarding treatment timing (12).

## Conclusion

Optimal patient selection is crucial for a favorable outcome even in cancer types with poor prognoses. Our case supports the further exploration of low-dose nivolumab in patients with MSI-high/TMB-high PDAC. This treatment has the advantages of relative safety and affordability.

## Data availability statement

The original contributions presented in the study are included in the article/**Supplementary Material**. Further inquiries can be directed to the corresponding author.

## Ethics statement

Ethical review and approval was not required for the study on human participants in accordance with the local legislation and institutional requirements. The patients/participants provided their written informed consent to participate in this study. Written informed consent was obtained from the individual(s) for the publication of any potentially identifiable images or data included in this article.

## Author contributions

Study design: SH-P, S-HY Data collection and analysis: SHP, B-BC, T-CK, J-CL Manuscript preparation: SH-P, S-HY.
