# Supplementary material for: Maintenance therapy of low-dose nivolumab, S-1, and leucovorin in metastatic pancreatic adenocarcinoma with a germline mutation of MSH6: A case report
Source: Front Immunol. 2022 Dec 13;13:1077840. doi: 10.3389/fimmu.2022.1077840 (PMC9792834; doi:10.3389/fimmu.2022.1077840)
Supplement: Supplementary file 1 [file Presentation_1.pptx]

## Slide 1
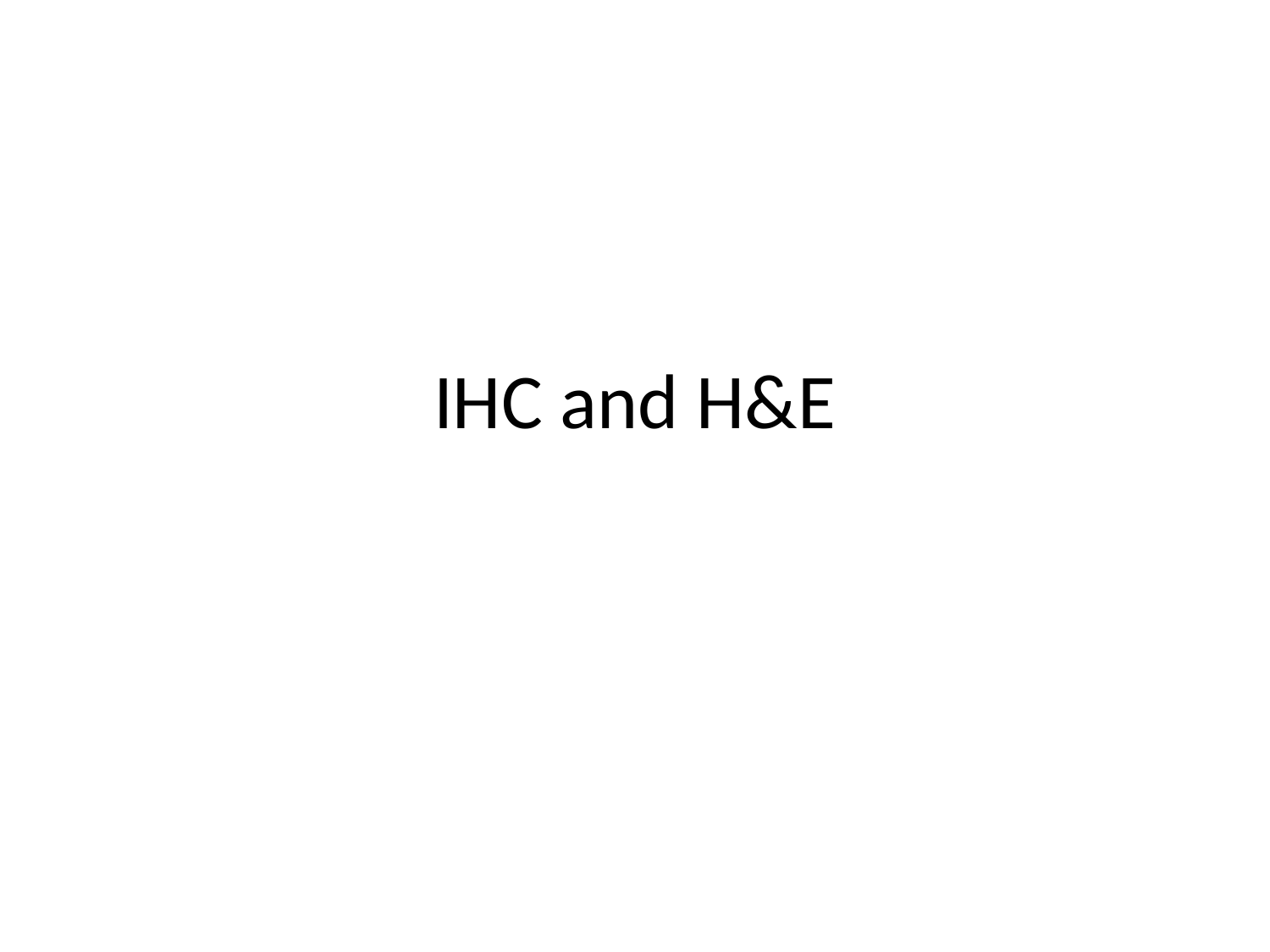

# IHC and H&E

## Slide 2
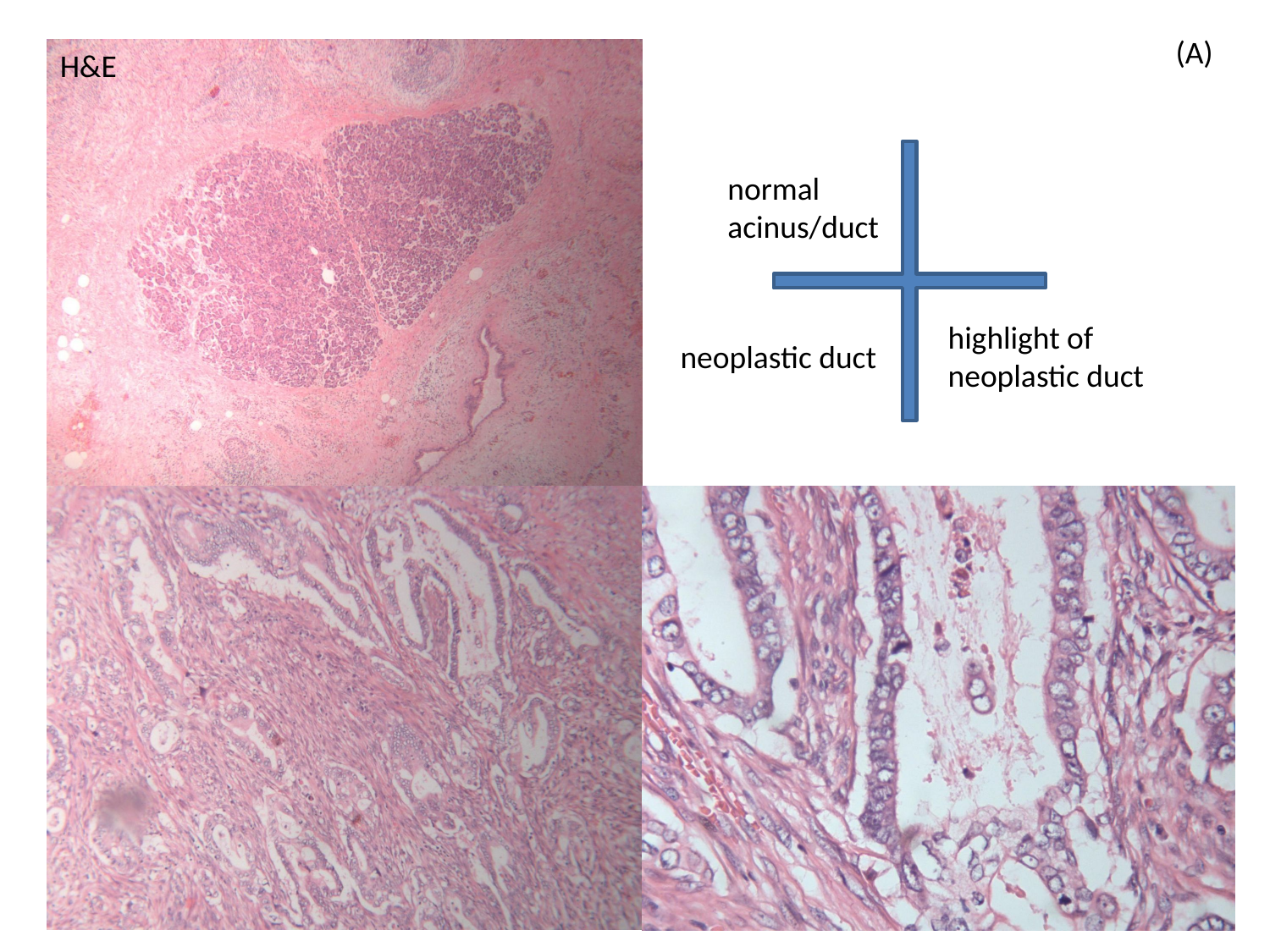

(A)
H&E
normal
acinus/duct
highlight of
neoplastic duct
neoplastic duct

## Slide 3
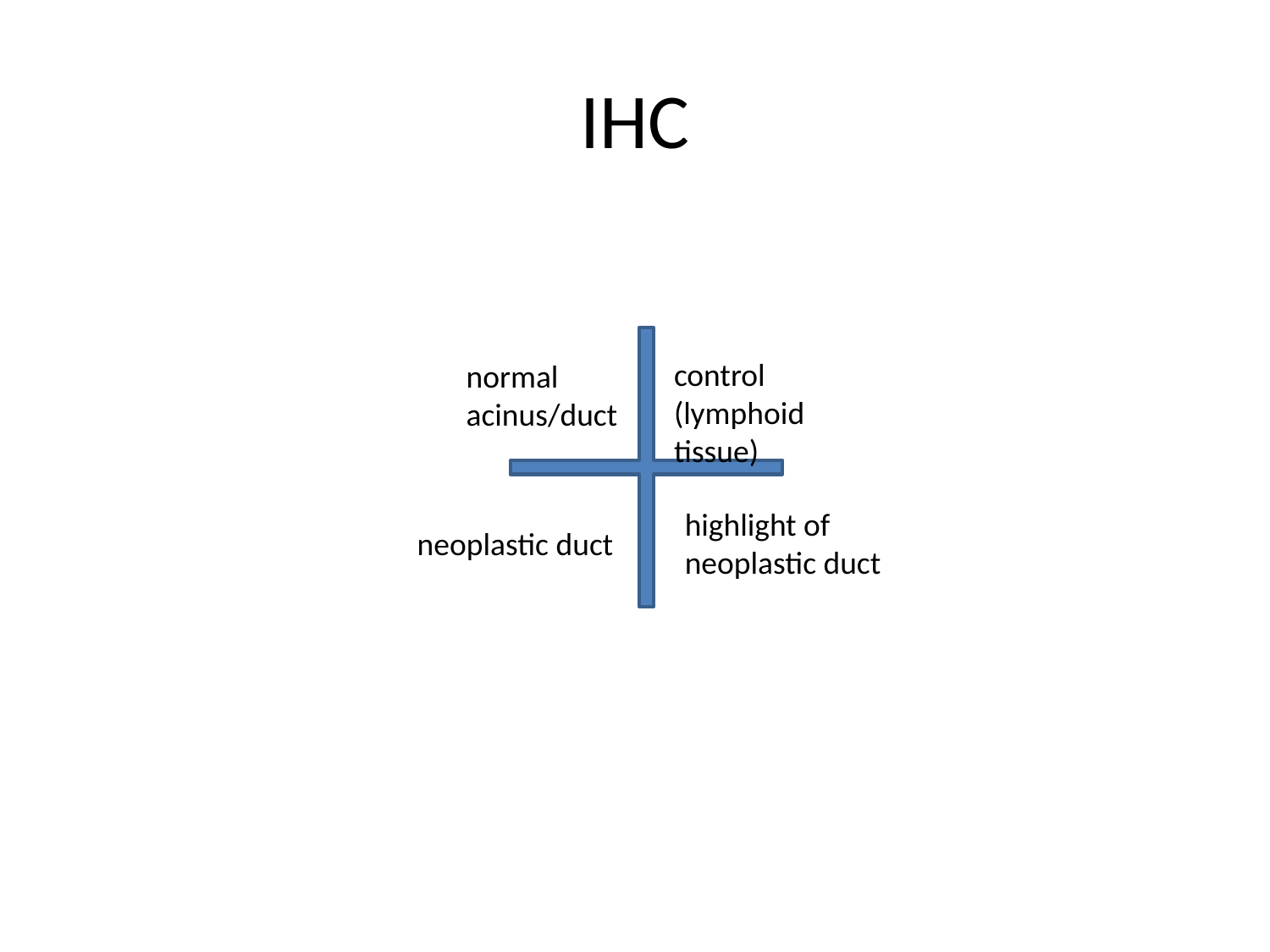

# IHC
control
(lymphoid tissue)
normal
acinus/duct
highlight of
neoplastic duct
neoplastic duct

## Slide 4
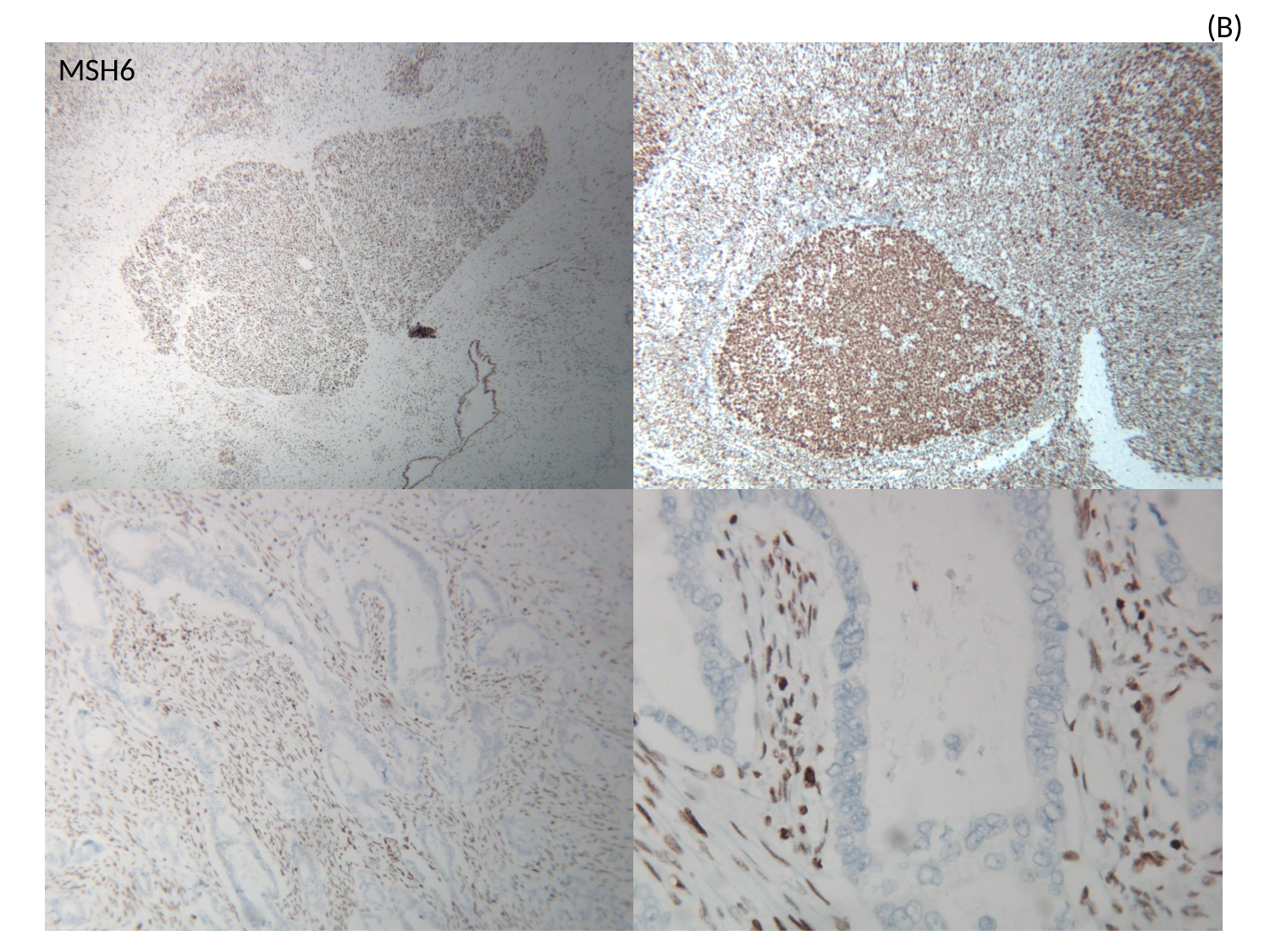

(B)
MSH6

## Slide 5
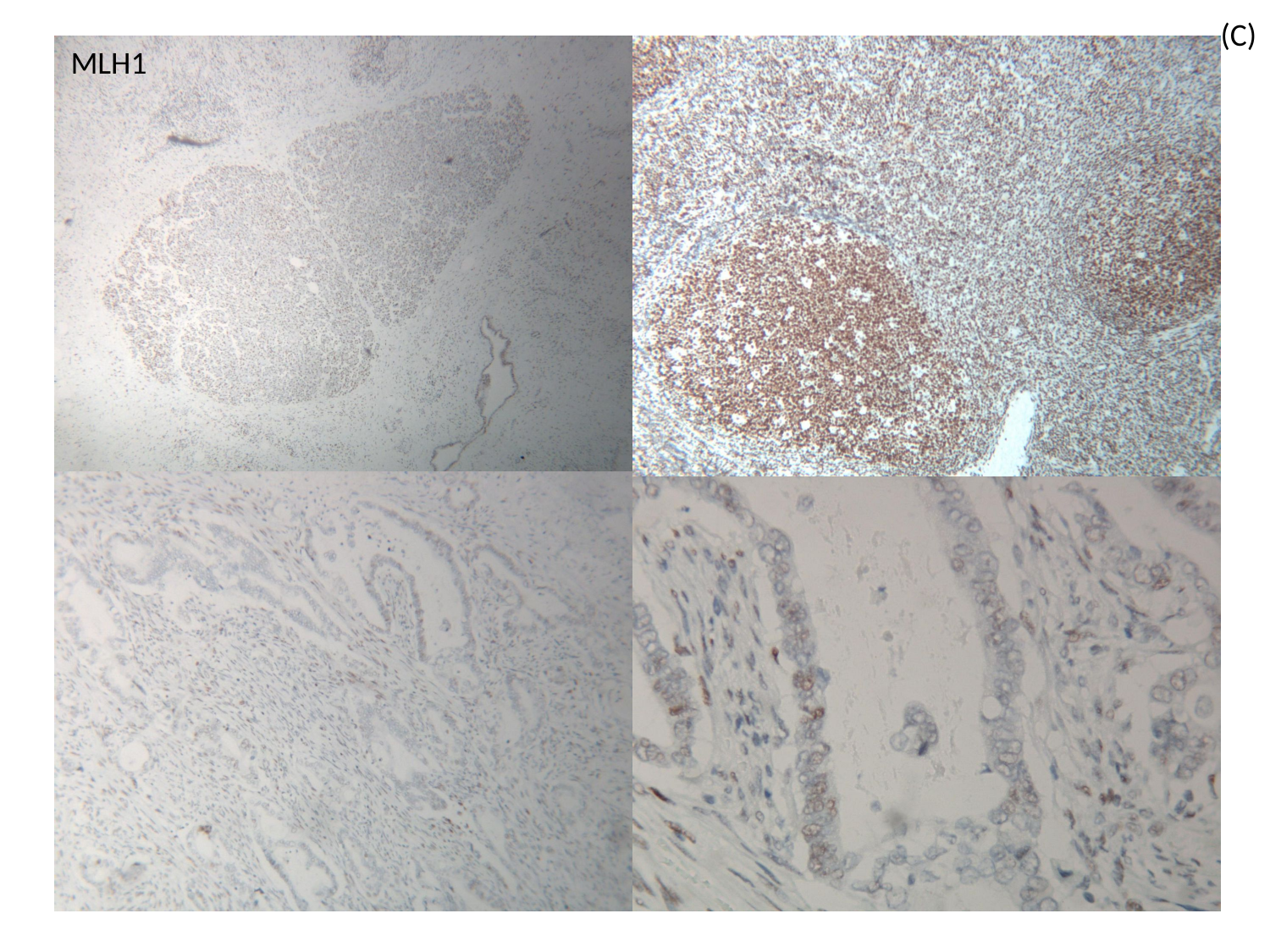

(C)
MLH1

## Slide 6
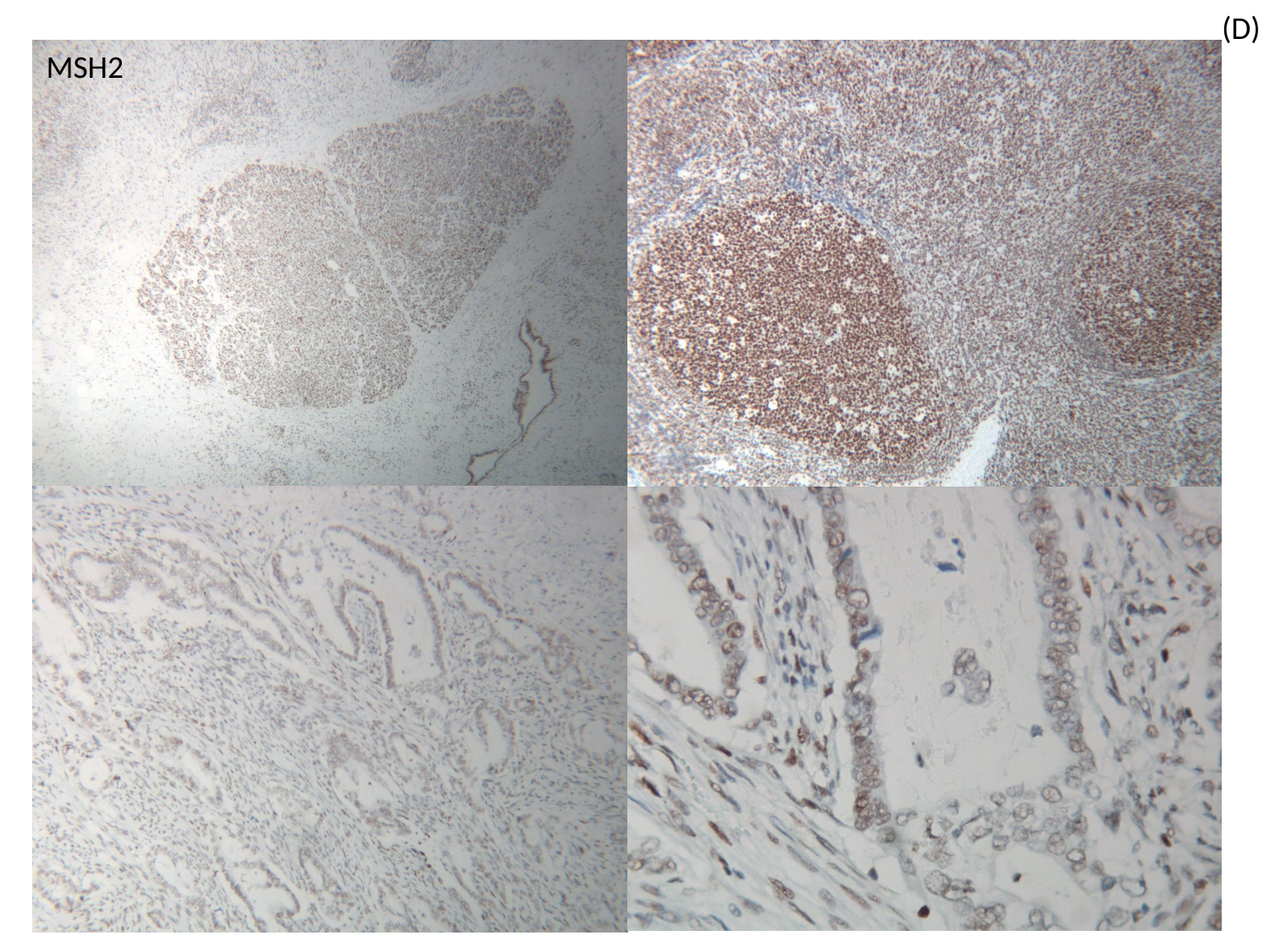

(D)
MSH2

## Slide 7
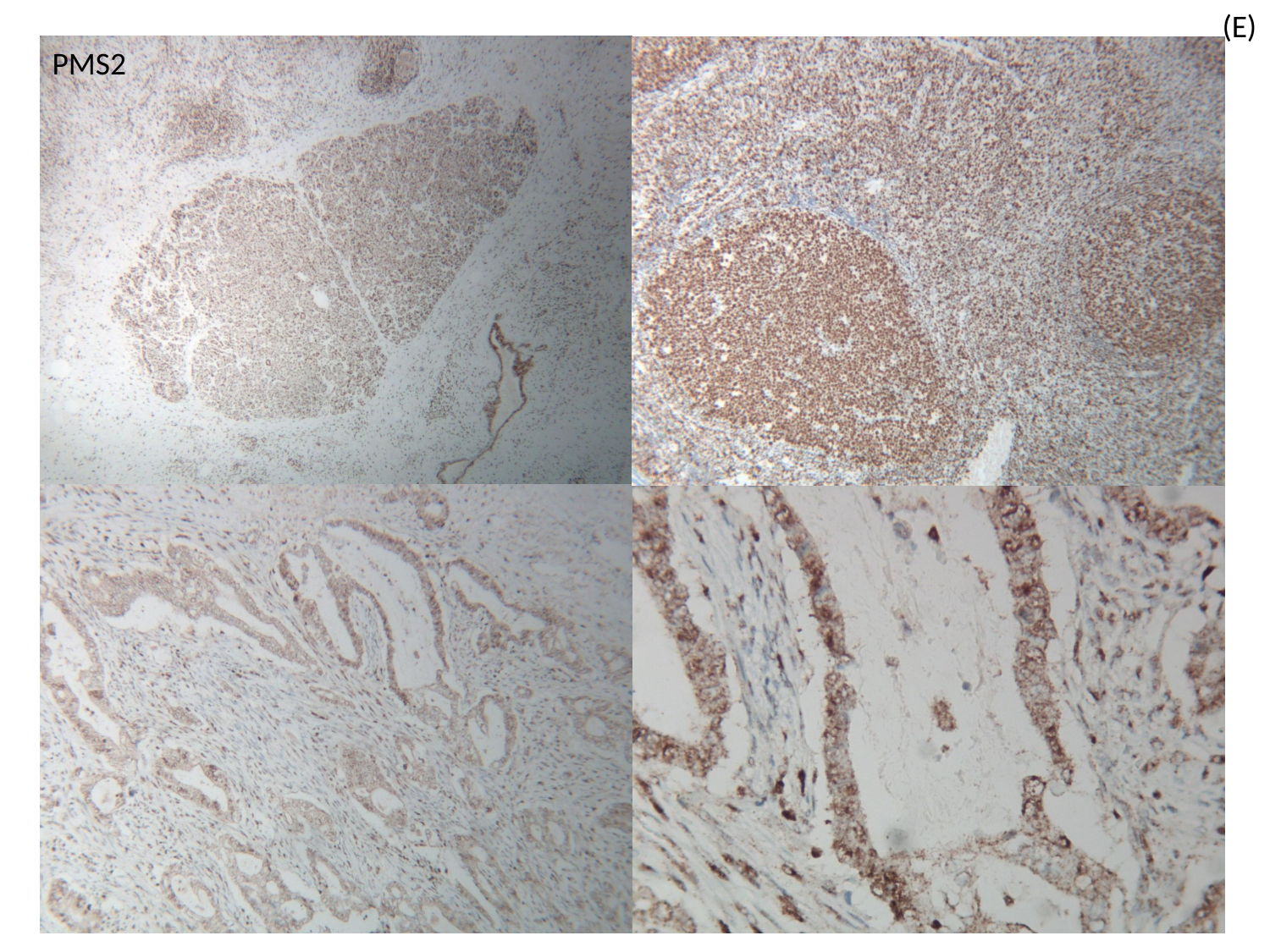

(E)
PMS2
