# Supplementary material for: Maintenance therapy of low-dose nivolumab, S-1, and leucovorin in metastatic pancreatic adenocarcinoma with a germline mutation of MSH6: A case report
Source: Front Immunol. 2022 Dec 13;13:1077840. doi: 10.3389/fimmu.2022.1077840 (PMC9792834; doi:10.3389/fimmu.2022.1077840)
Supplement: Supplementary file 2 [file Table_1.pdf]

Supplement Table 1 Additional genetic alterations

Gene	Alterations	Coverage	Allele frequency
CDKN2A	D84G	475	17.7%
CTCF	R377H	2295	20.3%
KMT2C	R208*	2844	16.4%
KMT2C	R904*	424	15.6%
KRAS	G12L	3824	17.6%
MAP2K4	R304*	1766	22.7%
PIK3CA	R108H	1463	15.9%
POLE	R2127*	3462	17.3%
TP53	R273H	1430	20.3%
MSH6	Y1006*	1967	51.4%

Heterozygous deletion: *BAP1, CDKN2A, MAP2K4, TP53, SMAD4, STK11, FLCN*

Specimen tumor purity: 39%
